# Promethin Is a Conserved Seipin Partner Protein

**DOI:** 10.3390/cells8030268

**Published:** 2019-03-21

**Authors:** Inês G. Castro, Michal Eisenberg-Bord, Elisa Persiani, Justin J. Rochford, Maya Schuldiner, Maria Bohnert

**Affiliations:** 1 Department of Molecular Genetics, Weizmann Institute of Science, Rehovot 7610001, Israel; ines.castro@weizmann.ac.il (I.G.C.); michal.eisenberg@weizmann.ac.il (M.E.-B.); 2 Rowett Institute and Aberdeen Cardiovascular and Diabetes Centre, University of Aberdeen, Aberdeen AB25 2ZD, UK; e.persiani@abdn.ac.uk; 3 Institute of Cell Dynamics and Imaging, University of Münster, Von-Esmarch-Str. 56, 48149 Münster, Germany; 4 Cells-in-Motion Cluster of Excellence (EXC 1003—CiM), University of Münster, 48149 Münster, Germany

**Keywords:** promethin, TMEM159, seipin, BSCL2, SPG17, lipid droplet, LD, lipodystrophy, LDO, adipogenesis

## Abstract

Seipin (BSCL2/SPG17) is a key factor in lipid droplet (LD) biology, and its dysfunction results in severe pathologies, including the fat storage disease Berardinelli-Seip congenital lipodystrophy type 2, as well as several neurological seipinopathies. Despite its importance for human health, the molecular role of seipin is still enigmatic. Seipin is evolutionarily conserved from yeast to humans. In yeast, seipin was recently found to cooperate with the lipid droplet organization (LDO) proteins, Ldo16 and Ldo45, two structurally-related proteins involved in LD function and identity that display remote homology to the human protein promethin/TMEM159. In this study, we show that promethin is indeed an LD-associated protein that forms a complex with seipin, and its localization to the LD surface can be modulated by seipin expression levels. We thus identify promethin as a novel seipin partner protein.

## 1. Introduction

Seipin (BSCL2/SPG17) is a widely expressed protein with a central role in fat storage. It is involved in the biogenesis of lipid droplets (LDs), fat storage organelles that are present in virtually all cell types, as well as in the development of mature adipocytes, specialized fat storage cells [1,2].

Since its identification in 2001 [3,4], seipin has received pronounced attention due to its importance for human health. Mutations in the seipin gene are responsible for the most severe subtype of congenital generalized lipodystrophy [5,6], also known as Berardinelli-Seip congenital lipodystrophy type 2 (BSCL type 2) [3]. Intriguingly, in addition to the role of seipin in lipodystrophy, mutations in the seipin gene are also responsible for a heterogeneous group of motor neuropathies collectively termed neuronal seipinopathies [4,7].

Seipin is a membrane-spanning protein that localizes to the endoplasmic reticulum (ER). It contains a conserved central domain comprising two transmembrane segments and a luminal loop region, as well as N- and C-terminal domains exposed to the cytosol [1,2,8,9]. The molecular mechanisms underlying the diverse seipin-related diseases as well as the exact molecular function of seipin in the cell are currently enigmatic. The neurological seipinopathies are generally autosomal dominant diseases, suggesting that they might be caused by a toxic gain-of-function of the mutated proteins, for example via their aggregation within the ER [10]. In contrast, BSCL type 2 is an autosomal recessive disease, indicating that it might result from seipin loss-of-function. Indeed, numerous studies reported dramatic alterations in LD maturation and morphology [1] as well as effects on adipocyte differentiation [11] in cells lacking seipin, indicating a key functional role of seipin in lipid storage. These observations have been further substantiated by the development of several mouse models of seipin loss-of-function, which recapitulate many of the metabolic defects observed in patients [12,13,14,15].

Seipin forms circular homo-oligomers [16,17]. The two recent cryo-EM structures of seipin from human [18] and Drosophila [19] suggest that these oligomers form ring-shaped complexes. These seipin oligomers have been reported to localize to so-called ER-LD contact sites [20,21,22], places where the two organelles are in very close proximity to each other and that play important roles in LD biogenesis from the ER [23]. However, as seipin is dispersed throughout the ER, the functional significance of this specific localization is currently unclear. In addition to forming homo-oligomers, seipin has been shown to be physically linked to several partner proteins, amongst them the phosphatidic acid phosphatase lipin 1, the acylglycerol-phosphate acyltransferase AGPAT2, the glycerol-3-phosphate acyltransferase GPAT3, the ER Ca^2+^-ATPase SERCA, the steaoryl-CoA desaturase 1 (SCD1), the reticulon-like protein REEP1, and the adaptor protein 14-3-3β [24,25,26,27,28,29,30]. Therefore, seipin has been suggested to act as a molecular scaffold that orchestrates the functions of numerous factors involved in lipid metabolism and LD biogenesis [25,27], and to regulate the traffic of phospholipids to LDs [18]. The identity of seipin partner proteins, as well as the molecular nature of their cooperation, might thus be crucial to understanding the function of seipin, and eventually the molecular etiology of seipin-related diseases.

The yeast seipin homolog has recently been found to cooperate with two LD proteins termed lipid droplet organization (LDO) proteins of 16 and 45 kDa (Ldo16 and Ldo45) [31,32]. Ldo16 and Ldo45 are derived from overlapping genes and are thus structurally related. The LDO proteins predominantly localize to a defined subpopulation of cellular LDs and determine their surface proteome and positioning within the cell via an unknown mechanism [31]. Remote homology searches identify a human protein termed promethin that has strong structural similarities to LDOs, indicating that promethin could represent the human homolog of LDOs [31].

In this study, we investigated human promethin and its relationship to seipin. We show that promethin transcripts go up during adipogenesis and that the promethin protein is localized to LDs. Moreover, we find that in yeast, promethin is localized to the same subpopulation of LDs as LDOs, and identify promethin as a novel seipin partner protein in human cells. Intriguingly, seipin recruits promethin away from LDs to the ER, pointing towards a dynamic interplay of the two components that could be important for the physiological role of seipin and for LD structure and function.

## 2. Materials and Methods

### 2.1. Mammalian Cell Culture and Plasmids

MCF7 cells were cultured in DMEM high glucose (4.5 g/L), supplemented with 10% FBS, 100 U/mL penicillin and 100 µg/mL of streptomycin. AML12 cells were cultured in DMEM:F12 medium, supplemented with 10% FBS, 1× ITS supplement (insulin, transferrin, and selenium) and 40 ng/mL dexamethasone.

C3H10T1/2 cells were cultured and induced to differentiate to adipocytes as in Reference [17].

HEK293 cells were cultured in DMEM containing 10% FBS and transiently transfected using calcium phosphate. Briefly, 2.5 mg DNA was diluted in 86 µL per well of 1× HBS adjusted to pH 7.05, followed by 5.1 μL of 2.5 M CaCl_2_. The DNA/CaCl_2_ mixture was incubated for 20 min at room temperature and subsequently added dropwise to the cells.

All cells were maintained at 37 °C and 5% CO_2_. MCF7 and AML12 cells were transfected using Lipofectamine 2000 (Invitrogen, Carlsbad, CA, USA) according to the manufacturer’s instructions. To induce LD accumulation, cells were treated with oleic acid (2 mol/mol Albumin, Merck, Kenilworth, NJ, USA) for 48–72 h at a final concentration of 200 µM. For immunofluorescence, cells were grown on 19 mm round glass coverslips in 12 well plates. For immunoprecipitation, cells were grown in 10 cm round dishes and processed 48 h after transfection.

Flag-Promethin was cloned from a pCMV6-AC-promethin-GFP plasmid (RG202226, Origene, Rockville, MD, USA) into the pEFIRES-P vector using restriction-free cloning and the Flag-tag sequence was introduced in the primer design. Seipin-myc long, short, and A212P plasmids have been previously described [33]. Briefly, the long and short isoforms of seipin were amplified and inserted in the pcDNA3.1mycHIS(A)-plasmid between the BamH1 and HindIII sites. The A212P plasmid was created by site-directed mutagenesis.

### 2.2. Yeast Growth, Strains, and Plasmids

*Saccharomyces cerevisiae* (from now on referred to as yeast) strains ERG6-mCherry (his3Δ1 leu2Δ0 met15Δ0 ura3Δ0 can1Δ::STE2pr-spHIS5 lyp1Δ::STE3pr-LEU2 ERG6-mCherry::NAT) and PDR16-mCherry (his3Δ1 leu2Δ0 met15Δ0 ura3Δ0 can1Δ::STE2pr-spHIS5 lyp1Δ::STE3pr-LEU2 PDR16-mCherry::NAT) were constructed using the pFA6a-Cherry-NAT plasmid [34], on the laboratory strain BY4741 [35], by a transformation method based on the usage of Li-acetate, PEG, and ssDNA [34,36,37]. For expression of human promethin in yeast cells, plasmid hPromethin-GFP (pRS426 backbone with human promethin-GFP under control of a GPD-promoter and CYC1-terminator) was constructed using restriction-free cloning.

### 2.3. RNA Extraction and Quantification

For the mRNA quantification, total RNA was extracted from differentiating adipocytes using the RNeasy mini kit (Qiagen, Hilden, Germany) following the manufacturer’s protocol. Equal quantities of RNA were DNase I treated (Sigma-Aldrich, St. Louis, MO, USA) then reverse transcribed with M-MLV reverse transcriptase, 5× reaction buffer, dNTPs and random primers (Promega, Madison, WI, USA). Real-time quantitative PCR was performed on the 7900HT system (Applied Biosystems, Foster City, CA, USA) or CFX384 Touch™ Real-Time PCR Detection System (BioRad, Hercules, CA, USA). NTC and NoRT controls were performed for every gene analyzed as in Reference [12]. The stable reference gene Ywhaz was used for normalization.

### 2.4. Immunofluorescence

MCF7 cells grown on glass coverslips were fixed 72 h after transfection with 4% paraformaldehyde, permeabilized with 0.2% Triton X-100 and blocked with 1% BSA. After blocking, cells were incubated with primary and secondary antibodies sequentially for 1 h, and finally with LipidTOX™ (Invitrogen, USA) for 45 min. Cells were washed 3× with PBS between all immunofluorescence steps. Antibodies used: Anti-promethin HPA063509 (Atlas Antibodies, Bromma, Sweden), anti-flag F1804 (Sigma-Aldrich, St. Louis, MO, USA), anti-Myc (9E10) sc-40 (Santa Cruz Biotechnology, Dallas, TX, USA).

### 2.5. Immunoprecipitation

MCF7 and AML12 cells were washed 3× in PBS and solubilized using lysis buffer (25 mM Tris HCl, pH 7.5, 150 mM NaCl, 0.5 mM EDTA) supplemented with 1% (*v*/*v*) NP-40 and protease inhibitors (complete Ethylenediaminetetraacetic acid [EDTA]-free cocktail (Roche), and 2 mM Phenylmethylsulfonyl fluoride (PMSF, Sigma-Aldrich)). Samples were clarified by centrifugation at 15.000× *g* for 15 min at 4 °C and incubated for 2 h at 4 °C with anti-FLAG M2 magnetic beads (Sigma-Aldrich). Beads were washed three times with lysis buffer supplemented with protease inhibitors and 0.1% (*v*/*v*) NP-40 and bound proteins were eluted by addition of 0.2 M glycine, pH 2.5. After neutralization by addition of 10% (*v*/*v*) 1 M Tris, pH 9.4, samples were subjected to SDS polyacrylamide gel electrophoresis (PAGE) and Western blotting using antibodies directed against FLAG (Sigma-Aldrich, St. Louis, MO, USA), Myc (ABCAM, Cambridge, UK), actin (ABCAM, Cambridge, UK), and ATF6 (Novus Biologicals, Centennial, CO, USA).

HEK293 cells were transiently transfected with plasmids encoding myc-tagged forms of either the long or short translations of seipin or seipin harboring the pathogenic A212P point mutation, as in Reference [17]. Following lysis, FLAG-tagged promethin was immunoisolated using anti-FLAG agarose beads (Sigma-Aldrich), washed in lysis buffer and subsequently eluted from beads by addition of 3xFLAG peptide (Sigma-Aldrich) in TBS (50 mM Tris–HCl, pH 7.4, 150 mM NaCl) before preparation in the loading dye, as in Reference [25]. Lysate and immunoprecipitated samples to be probed for seipin were not heated prior to loading to prevent aggregation. All other samples were heated to 95 °C for 5 min. Samples were separated by SDS-PAGE and transferred onto nitrocellulose membranes. Immunoblots were probed with antibodies to Myc (clone 4A6 Millipore), Flag (Sigma-Aldrich), or calnexin (ABCAM), followed by horseradish peroxidase (HRP)-linked secondary antibodies (Thermo Scientific, Waltham, MA, USA). Proteins were visualized using enhanced chemiluminescence.

Yeast cells grown to mid-logarithmic growth phase in synthetic minimal media (0.67% [*w*/*v*] yeast nitrogen base with ammonium sulfate, 2% [*w*/*v*] glucose, amino acid supplements) were harvested by centrifugation and washed in distilled water. Subsequently, cells were resuspended in lysis buffer (50 mM Tris HCl, pH 7, 150 mM NaCl, protease inhibitors (complete Ethylenediaminetetraacetic acid (EDTA)-free cocktail; Roche)), and single drops of cell suspensions were snap-frozen through dripping into liquid nitrogen. Frozen cells were ground using a ball mill (30 s at 30 Hz; Retsch, Haan, Germany). Ground cells were thawed and resuspended in lysis buffer supplemented with 1% digitonin (Sigma-Aldrich). Upon incubation at 4 °C for 1 h, samples were clarified using a centrifugation step (19.000 rpm in an SW41 swingout rotor for 30 min at 4 °C) and supernatants were incubated with a GFP-trap (Chromotek, Planegg-Martinsried, Germany) for 1 h at 4 °C. Beads were washed three times in lysis buffer and two times in PBS. Bound proteins were eluted through addition of 0.2 M glycine, pH 2.5, followed by neutralization with 10% (*v*/*v*) 1 M Tris, pH 9.4 and analysis by SDS PAGE and Western blotting using an anti-GFP antibody (ChIP Grade ab290, ABCAM, Cambridge, UK), antibodies directed against Sei1 and Ldb16 [32], and Histone H3 (ChIP Grade ab1791, ABCAM).

### 2.6. Microscopy of Cultured Mammalian Cells and Yeast

Mammalian cells were imaged using Zeiss LSM 780 and 800 confocal microscopes equipped with a 63×/1.4 NA (oil) objective (Carl Zeiss, Oberkochen, Germany), using the Diode 405 nm, Argon 488 nm, and He 543 nm laser lines. Image analysis and processing were performed using ImageJ v1.52 software.

Yeast cells were cultured over-night in synthetic minimal media (0.67% [*w*/*v*] yeast nitrogen base with ammonium sulfate, 2% [*w*/*v*] glucose, amino acid supplements) at 30 °C. Subsequently, cells were diluted and grown until reaching the mid-logarithmic phase. Cells were moved to glass-bottom 384-well microscope plates (Matrical Bioscience, Spokane, WA, USA) coated with Concanavalin A (Sigma-Aldrich). After 20 min, wells were washed twice with media to remove non-adherent cells. Yeast cells were imaged using a VisiScope Confocal Cell Explorer system (Visitron Systems, Puchheim, Germany) composed of a Zeiss Yokogawa spinning disk scanning unit (CSU-W1) coupled with an inverted Olympus IX83 microscope (Olympus, Tokyo, Japan). Single-focal-plane images were acquired with a 60× oil lens and were captured using a PCO-Edge sCMOS camera, controlled by VisiView software (GFP (488 nm) or RFP (561 nm)) (Visitron Systems, Puchheim, Germany).

## 3. Results

### 3.1. Promethin is an LD-associated Protein That Is Induced during Adipogenesis

**Figure 1:**
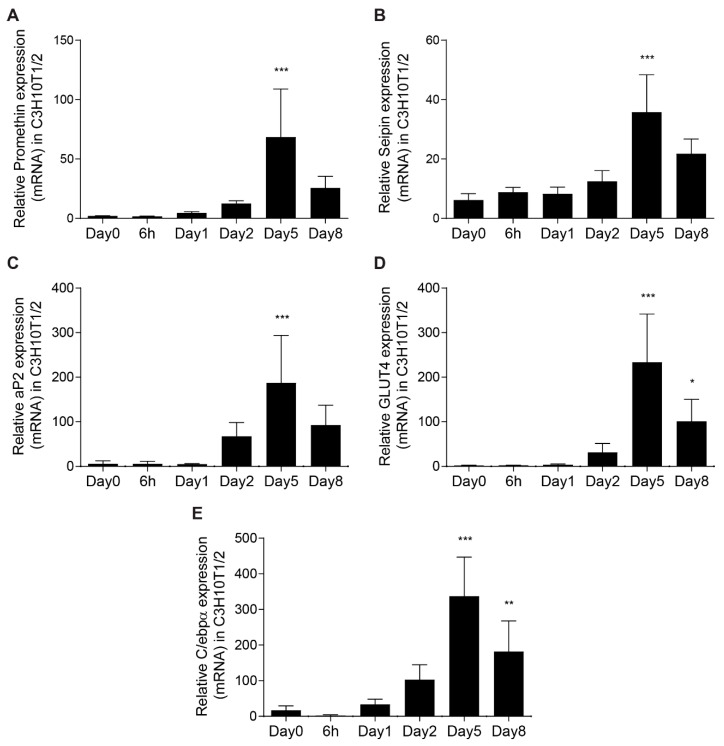
Promethin is upregulated during adipogenesis. (**A**) Promethin mRNA expression was determined in differentiating C3H10T1/2 cells, a model of adipogenesis. RNA was extracted at the time points shown and promethin expression was normalized to the housekeeping gene *Ywhaz*. Statistical analysis was performed with one-way ANOVA followed by Dunnett’s multiple comparison test. Statistically significant change in expression compared with that at Day 0 is indicated by: * *p* < 0.05, ** *p* < 0.01 and *** *p* < 0.001. Data are means ± SD, *n* = 4. Seipin (**B**), C/ebpα (**C**), aP2 (**D**) and Glut4 (**E**) mRNA expression was analyzed as described in (**A**). The induction of promethin and seipin mRNA expression correlated strongly during adipocyte development.

Seipin has been reported to be upregulated during the process of adipocyte development [33]. In order to investigate a possible cooperation between seipin and the potential LDO homolog promethin, we first determined promethin mRNA levels during adipogenesis using C3H10T1/2 mesenchymal stem cells. This revealed that promethin expression was strongly induced during adipogenesis, with expression peaking at day 5 (Figure 1A). Analysis of C/ebpα, aP2 and Glut4 mRNA expression was used to confirm efficient differentiation of these cells (Figure 1C–E). Interestingly, the induction of promethin expression correlated well with seipin expression during adipogenesis, indicating that both proteins are abundantly expressed in differentiated adipocytes (Figure 1B).

**Figure 2:**
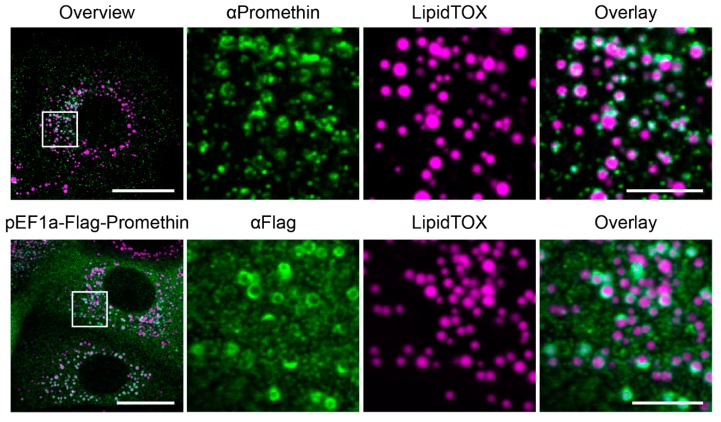
Promethin is an LD-associated protein. MCF7 cells treated with 200 µM oleic acid for 72 h were subjected to staining with the neutral lipid dye LipidTOX and immunofluorescence microscopy using an antibody directed against the C-terminus of human promethin (**top** row). MCF7 cells transfected with a plasmid for expression of promethin-Flag were subjected to the same procedure using an antibody against Flag (**bottom** row). Both native and expressed promethin localizes to lipid droplets (LDs). Scale bar, overview 20 µm; zoomed overlay, 5 µm.

This finding is consistent with a possible collaboration of promethin with seipin, and therefore prompted us to next analyze the subcellular distribution of promethin. A previous study had tentatively assigned promethin overexpressed in HEK293 cells to be cytosolic [38]. To have a better understanding of promethin localization, we analyzed endogenous, natively expressed promethin. Immunostaining with an antibody directed against a C-terminal peptide of promethin in the breast cancer cell line MCF7 revealed a dispersed pattern in cells grown in regular media, as previously reported (Figure S1). However, treatment with oleic acid to induce LD accumulation resulted in the localization of promethin to a circular pattern throughout the cytosol (Figure 2, top row), indicating that the distribution of promethin is affected by the metabolic state of the cell.

Utilizing the LD dye LipidTOX, we found that these promethin positive structures are co-localizing with LDs (Figure 2, top row), suggesting that promethin is either an LD surface protein or that it localizes to subdomains of the ER that are in very tight contact with LDs. Interestingly, some LDs showed a strong promethin signal, while part of the cellular LD population had very low promethin signal at its surface (Figure 2, top row). This suggests that, similar to the LDO components in yeast cells, promethin is concentrated on a subpopulation of the cellular LD pool [31]. Additionally, promethin also appears to localize to puncta in the cell that are not associated with any of the stained lipid droplets. These could represent ER subdomains or LD biogenesis sites. However, further work will be necessary to resolve this question.

We next transfected MCF7 cells using a Flag-tagged promethin construct and performed immunofluorescence microscopy using an anti-Flag antibody. This approach resulted in elevated promethin levels, hence, some cells had too much of a cytosolic background to see the LD localization. However, focusing on cells with lower expression levels, we found that promethin also localized to LDs during growth on oleic acid (Figure 2, bottom row).

As both human promethin and yeast LDO proteins are enriched on a fraction of the cellular LD pool, we wanted to test whether their preference for specific LDs has been conserved in evolution. Therefore, we expressed a GFP-tagged variant of human promethin in yeast cells that expressed LD marker proteins fused to an mCherry tag. GFP-tagged promethin localized to only a subset of the entire LD pool labeled by Erg6-mCherry (a resident of all LDs) (Figure S2A, top row). Similarly to LDOs, this subset of LDs was characterized by localization of the subpopulation marker Pdr16-mCherry [31] (Figure S2A, bottom row). This similar distribution pattern of human promethin and yeast LDOs supports a functionally conserved role for these proteins.

### 3.2. Promethin Is a Novel Seipin Partner Protein

**Figure 3:**
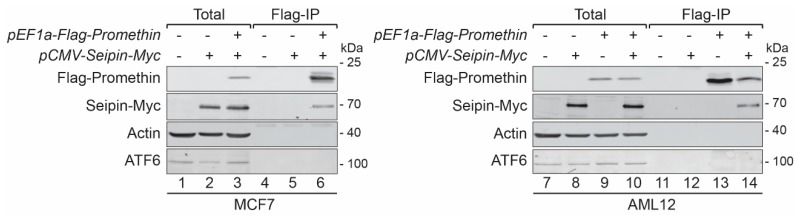
Promethin forms a complex with the LD biogenesis protein seipin. MCF7 and AML12 cells transfected with plasmids for expression of Flag-promethin and/or seipin-Myc were treated with 200 µM oleic acid for 48 h and subjected to immunoprecipitation using agarose beads coupled to Flag-antibodies, and eluted proteins were analyzed by SDS-PAGE and Western blotting. Seipin is specifically co-isolated with promethin, while the endoplasmic reticulum (ER) membrane protein ATF6 is not detectable in immunoprecipitations.

In yeast, the seipin complex is formed by two components: Sei1 and Ldb16 [39], and we have previously shown that the LDO proteins interact with this complex. To address if this is also true for human promethin and seipin, we co-expressed Flag-tagged promethin with Myc-tagged seipin in MCF7 cells and performed immunoprecipitations using antibodies directed against the Flag-tag. We found that seipin was specifically co-isolated with promethin, while the abundant ER membrane protein ATF6 was not co-purified (Figure 3, lane 6). Furthermore, we obtained similar results using the hepatocyte cell line AML12 (Figure 3, lane 14), showing that seipin and promethin are closely linked in different cell types. As expected by the similar subcellular distribution pattern, human promethin expressed in yeast also efficiently co-isolated the yeast seipin components, Sei1 and Ldb16 (Figure S2B, lane 4).

The BSCL2 gene encodes two isoforms of seipin, a full length, long form (seipin-L), and a short form (seipin-S) that lacks the first 64 amino acids [8]. Hence, we also examined the interaction of promethin with seipin-L and seipin-S, as well as a pathogenic mutant form of seipin harboring the single amino acid substitution A212P (seipin-A212P) identified from patients with generalized lipodystrophy [3]. When co-expressed in HEK293 cells, we found that promethin efficiently co-purified all the above isoforms, suggesting that the interaction is a basic property of seipin (Figure S3, lanes 14–16). In summary, promethin and seipin are partner proteins.

### 3.3. Promethin Subcellular Localization Is Modulated by Seipin

**Figure 4:**
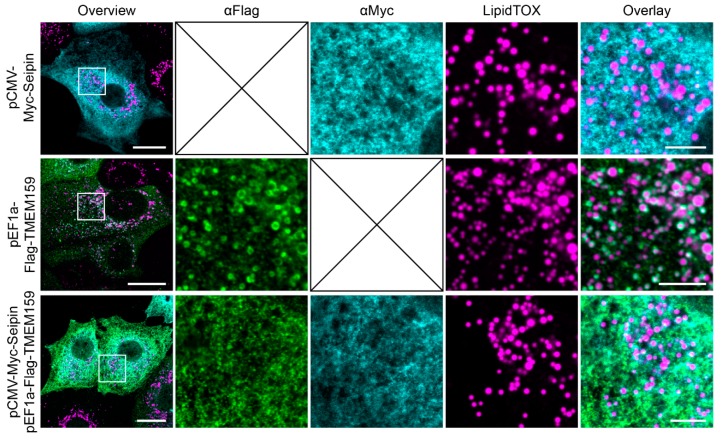
Seipin modulates promethin subcellular localization. MCF7 cells transfected with plasmids for expression of Flag-promethin and/or seipin-Myc were treated for 72 h with 200 µM oleic acid. Subsequently, LDs were stained with the neutral lipid dye LipidTOX and immunofluorescence microscopy was performed using antibodies against Flag and Myc. Seipin expression results in the loss of promethin from LDs and re-localization to the ER. Scale bar, overview 20 µm; zoomed overlay, 5 µm.

As seipin was efficiently co-isolated with promethin, we wanted to determine the spatial relationship of the two components within the cell. Therefore, we expressed Flag-promethin, seipin-Myc, or both proteins simultaneously in MCF7 cells and performed immunofluorescence microscopy. When expressed separately, promethin decorated the surface of LDs as in the previous experiments (Figure 2; Figure 4, middle row), and seipin was found in a reticular distribution (Figure 4, top row), consistent with numerous previous reports that identified seipin as an ER protein [1,2,11,40,41]. Strikingly, co-expression of seipin dramatically altered the subcellular distribution of promethin. Upon seipin co-expression, promethin lost its circular pattern on the LD surface, and instead showed a reticular distribution similar to seipin (Figure 4, bottom row). Importantly, this re-distribution was not due to a loss of LDs, which were still present upon co-expression of promethin and seipin (Figure 4, bottom row). These results lead us to conclude that seipin levels modulate promethin localization within the cell, with high seipin levels resulting in the re-distribution of promethin from LDs to the ER. Since our immunoprecipitation experiments demonstrate that these two proteins form a complex, we suggest this effect to be mediated by direct binding of the two proteins. However, it remains possible that seipin overexpression affects promethin localization in a different, potentially indirect way, and further studies are required to reveal the exact nature of the interaction between these two proteins.

## 4. Discussion

In this study, we have identified promethin as a novel partner protein of seipin, a protein linked to several human diseases that has a key role in LD biology. Seipin is specifically co-isolated in promethin immunoprecipitations, indicating that the two proteins form a complex. Intriguingly, while promethin localizes to LDs under standard conditions, overexpression of seipin results in a striking re-distribution of promethin to the entire ER. We conclude that promethin and seipin are linked to each other, with seipin dynamically modulating promethin distribution in the cell.

Whether promethin is a genuine LD surface protein or whether it sits on ER membranes which are closely associated with LDs is still to be determined. Secondary structure predictions indicate that promethin contains a central α-helical hydrophobic domain (76 amino acids in length in both human and mouse), as well as hydrophilic N- and C-terminal domains [31]. This structure suggests that if promethin is localized to the LD surface, the hydrophobic domain might be integrated into the hydrophobic LD core, while the hydrophilic N- and C-terminal stretches are likely exposed to the cytosol. As overexpression of seipin results in efficient relocation of promethin to the ER, promethin might have the potential to alternatively be inserted into the phospholipid bilayer of the ER. Such an ability to localize to both organelles is consistent with current models of LD protein biogenesis that suggest that many LD proteins undergo a two-step biogenesis process, in which they are first transiently integrated into the ER membrane, before migrating over to the LD [42].

Promethin is a poorly characterized protein, and its exact molecular role remains to be determined. How could the expression level of seipin have such a dramatic effect on promethin subcellular localization? One possible explanation would be that promethin exists in two pools, an LD-localized pool independent of seipin, and a seipin-dependent pool within the ER. In such a model, seipin overexpression would result in a depletion of the LD pool of promethin by ER-localized seipin binding to all promethin molecules. Based on the recently published seipin structures, a model for seipin function has been proposed, in which the seipin complex operates in two functional modes during biogenesis of LDs from the ER. When in an initial “ER scanning mode”, seipin rings were suggested to move freely through the ER membrane and utilize hydrophobic α-helices to screen for membrane packing defects indicative of small neutral lipid lenses between its bilayer leaflets, which are believed to represent early stages of LD biogenesis. In this model, seipin subsequently adopts an “LD anchoring mode”, where the seipin ring reorganizes to stabilize growing LDs on the ER membrane and prevents premature severing of the connection between the emerging LD and its parent organelle [19,43]. Our observations are consistent with promethin having a preference for the ER-localized scanning mode seipin complexes. It will be interesting to assay, in the future, if promethin has a role in the conformational transition between the two seipin forms.

Alternatively, it is conceivable that seipin has a direct role in promethin biogenesis, possibly being involved in the handover of promethin from the ER to LDs. In this model, overexpressed seipin could trap promethin in the ER at an intermediate step of such a two-step biogenesis pathway.

Intriguingly, it has been found that promethin is strongly expressed in liver cells in response to overexpression of peroxisome proliferator-activated receptor γ1 (PPARγ1), a master regulator of lipid storage [38]. PPARγ1 overexpression in the liver results in a particular type of hepatic steatosis characterized by expression of adipocyte-specific and lipogenesis-related genes [38], pointing towards a possible role of promethin in lipid storage, LD biogenesis and/or adipogenesis in collaboration with seipin. 

## 5. Conclusions

We conclude that promethin is a partner protein of the LD formation component seipin. Moreover, we find that the subcellular distribution of promethin is dynamically regulated between LD and ER associated localizations by the levels of seipin. This adds to our understanding of the molecular roles of seipin, an important protein for the regulation of LD biology and adipose tissue function. It also highlights a novel potential role for promethin in these processes. Future work will be required to understand the exact functional interplay of promethin with seipin, and will hopefully help us unravel the pathological processes underlying BSCL type 2, as well as neuronal seipinopathies.

## Supplementary Materials

The following are available online at https://www.mdpi.com/2073-4409/8/3/268/s1. Figure S1: Promethin localization in MCF7 cells in the absence of oleic acid; Figure S2: Human promethin is linkec to the yeast seipin complex; Figure S3: Promethin and seipin interaction is isoform independent.
