# Supplementary material for: Promethin Is a Conserved Seipin Partner Protein
Source: Cells. 2019 Mar 21;8(3):268. doi: 10.3390/cells8030268 (PMC6468817; doi:10.3390/cells8030268)
Supplement: Supplementary file 1 [file cells-08-00268-s001.pdf]

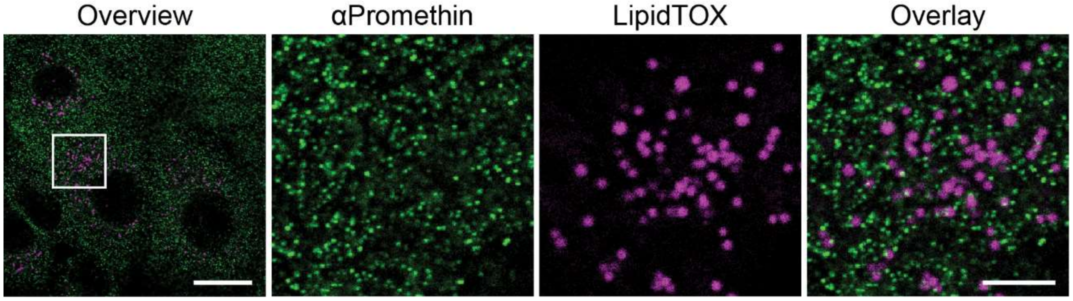

Figure S1: Promethin localization in MCF7 cells in the absence of oleic acid. MCF7 cells were subjected to staining with the neutral lipid dye LipidTOX and immunofluorescence microscopy using an antibody directed against the C-terminus of human promethin. In the absence of oleic acid, endogenous promethin presents a dispersed pattern. Scale bar, overview 20 µm; zoomed overlay 5 µm.

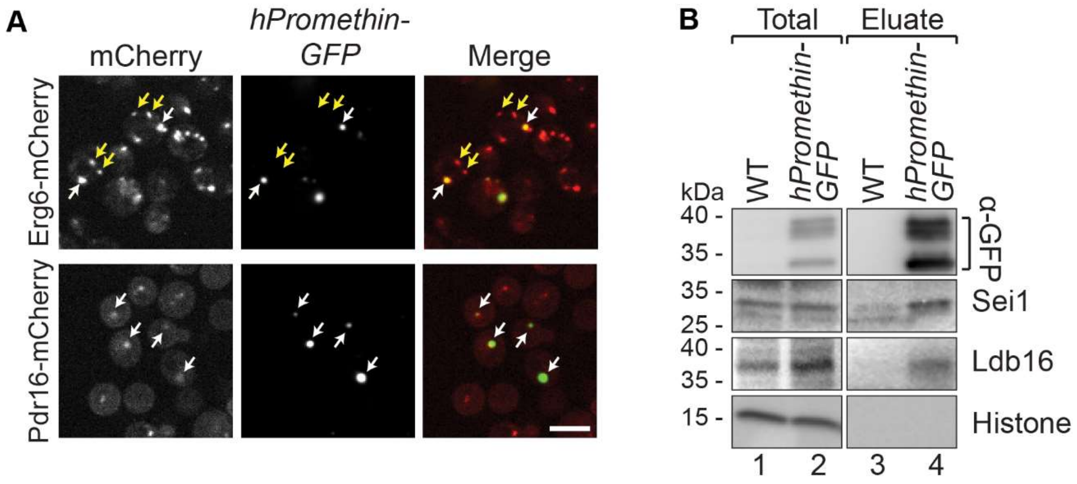

Figure S2: Human promethin is linked to the yeast seipin complex. (A) Human promethin-GFP expressed in yeast localizes just to a subpopulation of LDs marked by the general LD marker Erg6-Cherry (top, white arrows) and fully co-localizes with the subpopulation marker Pdr16-Cherry (bottom). Scale bar, 5µm. (B) Human promethin fused to GFP was overexpressed in yeast. Cells were subjected to immunoprecipitation using a GFP-trap, bound proteins were eluted using glycine, pH 2.5, and samples were analyzed by SDS-PAGE and western blotting. Human promethin co-isolates the yeast seipin proteins Sei1 and Ldb16.

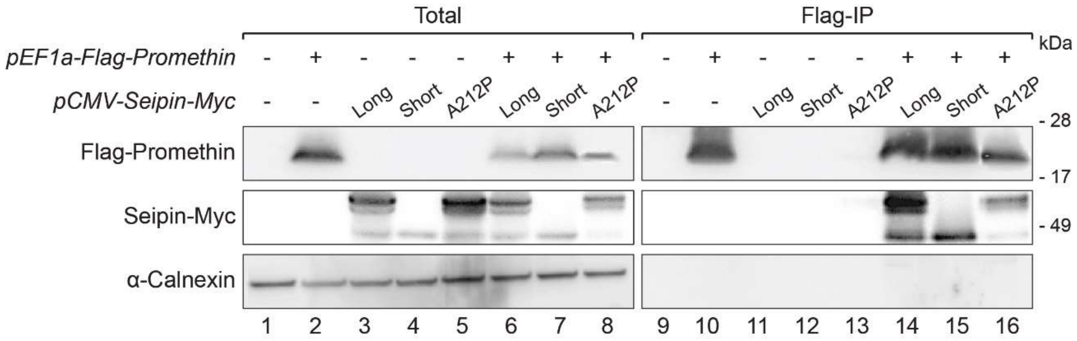

Figure S3: Promethin and seipin interaction is isoform independent. Myc tagged forms of the long and short translations of seipin, or an A212P mutant form of seipin were co-expressed with Flag-promethin in HEK293 cells as indicated. Cells were subjected to immunoprecipitation using an antibody directed against Flag. The ER membrane resident protein calnexin was blotted to show equal loading of lysates and also serves as a negative control for the immunoprecipitations. All tested seipin variants were co-isolated with promethin.
